# ICSI with surgically retrieved sperm in azoospermia: protocol for a systematic review and meta-analysis of reproductive, perinatal, long-term, and paternal outcomes

**DOI:** 10.1186/s13643-025-03021-9

**Published:** 2025-12-10

**Authors:** Panagiotis Tsiartas, Rocio Montejo, Stavros I. Iliadis, Francisco Guillen‑Grima

**Affiliations:** 1 Department of Clinical Science, Intervention and Technology (CLINTEC), Karolinska Institute, 171 77 Stockholm, Sweden; 2 Department of Obstetrics and Gynecology, Sahlgrenska University Hospital, 413 46, Gothenburg, Sweden; 3 Institute of Clinical Sciences, Sahlgrenska Academy, University of Gothenburg, 413 46, Gothenburg, Sweden; 4 Department of Women’s and Children’s Health, Uppsala University, 751 85 Uppsala, Sweden; 5 Department of Preventive Medicine, Clínica Universidad de Navarra, 310 08 Pamplona, Spain; 6 Healthcare Research Institute of Navarre (IdiSNA), 310 08 Pamplona, Spain; 7 CIBER in Epidemiology and Public Health (CIBERESP), Institute of Health Carlos III, 469 80 Madrid, Spain; 8 Department of Health Sciences, Public University of Navarra, 310 08 Pamplona, Spain

**Keywords:** Azoospermia, ICSI, Surgically retrieved sperm, TESE, PESA, Micro‑tese, Live birth, Perinatal outcomes, Meta-analysis

## Abstract

**Background:**

Azoospermia affects ~ 1% of men and represents a major cause of severe male infertility. Intracytoplasmic sperm injection (ICSI) with surgically retrieved sperm from the testis or epididymis enables biological fatherhood in obstructive (OA) and non-obstructive azoospermia (NOA) patients. However, comparative studies versus ejaculated sperm remain fragmented, particularly for neonatal and long-term offspring outcomes. This study aims to address the existing evidence gap regarding the outcomes of ICSI cycles using surgically retrieved versus ejaculated sperm in men with azoospermia by evaluating reproductive, perinatal, long-term offspring, and paternal outcomes.

**Methods:**

A systematic review and meta-analysis will be conducted following the PRISMA-P and PRISMA 2020 guidelines. Eligible studies will include randomized and non-randomized comparative designs of ICSI using surgically retrieved versus ejaculated sperm as single-arm studies for outcomes not applicable to ejaculated sperm. The primary outcome is live birth per oocyte pick-up or per started cycle. The secondary outcomes include reproductive, perinatal, long-term offspring, and paternal outcomes. Searches will be conducted in PubMed, Embase, Scopus, CENTRAL, Web of Science, and ProQuest, supplemented by trial registries, conference proceedings, and grey literature from 1990 onward. The risk of bias will be assessed with RoB 2 and ROBINS-I; certainty of evidence will be assessed with GRADE. Random-effects meta-analyses with restricted maximum likelihood estimation and Hartung-Knapp-Sidik-Jonkman adjustment will be applied, with rare-event models explored as sensitivity analyses.

**Discussion:**

This protocol describes a systematic approach to evaluate the efficacy and safety of ICSI via surgically retrieved sperm compared with ejaculated sperm, with a particular focus on filling current evidence gaps in perinatal, offspring, and paternal outcomes. The results will contribute to evidence-based counseling, clinical practice, and guideline refinement.

**Systematic review registration:**

PROSPERO CRD420251142427.

**Supplementary Information:**

The online version contains supplementary material available at 10.1186/s13643-025-03021-9.

## Background and rationale

Azoospermia, defined as the complete absence of spermatozoa in the ejaculate, affects approximately 1% of all men and accounts for approximately 10% to 15% of cases of male infertility [1, 2]. It is classified as either obstructive azoospermia (OA), which is caused by reproductive tract blockage, or non-obstructive azoospermia (NOA), which is caused by impaired spermatogenesis. NOA is the predominant form, representing nearly 60% of cases and posing distinct therapeutic challenges [3].

Advances in assisted reproductive technology (ART), particularly intracytoplasmic sperm injection (ICSI), which was first reported in 1992 [4], have enabled men with azoospermia to achieve biological parenthood. Surgical sperm retrieval techniques including testicular sperm aspiration (TESA), testicular sperm extraction (TESE), microdissection testicular sperm extraction (micro-TESE), and epididymal methods, such as percutaneous epididymal sperm aspiration (PESA) and microsurgical epididymal sperm aspiration (MESA), expand options by facilitating epididymal or testicular sperm use. Micro–TESE, introduced in 1999, improved targeted retrieval while minimizing testicular tissue loss [5].

Despite widespread clinical use, uncertainty persists regarding the effectiveness and safety of ICSI with surgically retrieved sperm compared with ejaculated sperm. Most observational studies [6–14] and prior reviews [15–17] emphasize fertilization and pregnancy outcomes, whereas data on live birth, neonatal outcomes, long‑term offspring health, and paternal harms (e.g., retrieval failure, perioperative complications) remain limited. Evidence fragmentation by azoospermia type and sperm source further impedes clinical guidance.

A prospectively registered, methodologically rigorous systematic review and meta-analysis is warranted to synthesize reproductive, perinatal, long-term offspring, and paternal outcomes and inform patients, clinicians, and policymakers.

## Aim

To compare the outcome of ICSI cycles using surgically retrieved sperm versus ejaculated sperm among men with azoospermia and to evaluate reproductive, perinatal, long-term offspring, and paternal outcomes across randomized controlled trials, quasi-experimental studies, and observational designs.

## Methods

### Registration

The methodological approach of this review will follow the Cochrane Handbook for Systematic Reviews of Interventions (version 6.5) [18]. Reporting will follow PRISMA-P [19] and PRISMA 2020 guidelines [20] (Additional file 1) and is registered in the International Prospective Register of Systematic Reviews (PROSPERO, ID: CRD420251142427, registered 22 September 2025).

### Participants

Individuals or couples undergoing ART in which the male partner was diagnosed with OA or NOA and autologous oocytes were used.

### Intervention

ICSI using surgically retrieved sperm (TESA, TESE, micro‑TESE, PESA, or MESA), fresh or frozen.

### Comparator

ICSI using ejaculated sperm.

### Primary outcome

Live birth rate per oocyte pick‑up or per started cycle. This denominator reflects the overall effectiveness of the ICSI treatment strategy, encompassing failed sperm retrieval, fertilization failure, and cycle cancellation, and provides the most comprehensive and patient-centered estimate of treatment success. In contrast, live birth per embryo transfer reflects implantation efficiency but excludes cycles that do not reach transfer, potentially overestimating success rates; it will therefore be analyzed as a secondary outcome.

### Secondary outcomes

Fertilization, biochemical/clinical/ongoing pregnancy, miscarriage, multiple pregnancy, live birth per embryo transfer, and cumulative live birth rates; perinatal outcomes (gestational age, preterm birth, birth weight, congenital anomalies, perinatal mortality, neonatal intensive care unit (NICU) admission); long-term offspring outcomes (growth, neurodevelopment, cognition, education, psychiatric, cardiometabolic); and paternal outcomes (retrieval failure, perioperative complications).

### Exclusion criteria

The exclusion criteria for this systematic review and meta-analysis are as follows: (1) donor oocyte cycles; (2) mixed patient cohorts in which outcomes for individuals with azoospermia cannot be separately identified; (3) use of round spermatid injection (ROSI), intracytoplasmic morphologically selected sperm injection (IMSI), physiological ICSI (PICSI), or magnetic-activated cell sorting (MACS) as the primary intervention; and (4) use of conventional in vitro fertilization (IVF).

### Study design

This review will include randomized controlled trials, quasi-experimental studies, prospective and retrospective cohort studies, and case-control studies providing comparative data relevant to the research question. Non-comparative studies will also be included for paternal outcomes inherent to surgical sperm retrieval that cannot be directly compared with ejaculated sperm. The following study designs will be excluded: case series without comparator groups, registry-only cohorts lacking clinic-based ascertainment (i.e., without direct outcome verification by the treating fertility clinic), conference abstracts lacking sufficient methodological detail, narrative reviews, scoping reviews, and other non-comparative study designs.

### Information sources and search strategy

We will search PubMed, Embase (Ovid), Scopus, the Cochrane Central Register of Controlled Trials (CENTRAL), Web of Science, and ProQuest Dissertations and Theses Global [18]. Additional sources will include ClinicalTrials.gov [21], the WHO International Clinical Trials Registry Platform (ICTRP) [22], and BASE (Bielefeld Academic Search Engine) for grey literature [23]. Conference proceedings and meeting abstracts will also be captured through the Web of Science and Scopus. The searches will cover literature published from 1990 to the present, without language restrictions. We selected 1990 as the starting point because it corresponds to the introduction and subsequent clinical implementation of ICSI. The initial search strategy will be developed by the review team in collaboration with an experienced medical librarian. The final strategy will then undergo peer review using the PRESS 2015 checklist [24]. The literature search will be focused on studies including men with azoospermia. To increase sensitivity, related search terms (e.g., cryptozoospermia, hypospermatogenesis, low sperm count, oligoasthenoteratozoospermia, oligozoospermia) will also be included to capture studies in which azoospermic men were represented, even if the term “azoospermia” was not explicitly mentioned. Only studies including azoospermic men will be retained for the final analysis. A full draft search strategy for PubMed is provided in Additional file 2. The full strategies used will be provided in the supplementary files and archived on the Open Science Framework (OSF).

### Study selection

Screening and study management will be conducted at Covidence systematic review software (Veritas Health Innovation, Melbourne, Australia). Available at www.covidence.org. Two reviewers will independently screen titles/abstracts and full texts (with blinding enabled during title/abstract screening). Disagreements will be resolved by discussion, with arbitration by a third reviewer when necessary. Inter-rater reliability will be quantified via Cohen’s κ statistic, and reasons for full-text exclusion will be documented. The study selection process will be summarized in a PRISMA 2020 flow diagram [20].

### Data extraction

Data will be extracted independently and in duplicate via piloted forms [18]. The extracted information will include the study design, participant and intervention characteristics, and all prespecified outcomes. When data are missing or unclear, the study authors will be contacted for clarification [18]. If only medians and interquartile ranges or ranges are reported, means and standard deviations will be estimated via validated methods [25–28].

### Risk of bias in individual studies

The risk of bias will be assessed in a standardized manner by two independent reviewers. For randomized controlled trials, we will use the Revised Cochrane Risk of Bias tool (RoB 2), which evaluates bias arising from the randomization process, deviations from intended interventions, missing outcome data, measurement of outcomes, and selection of the reported results [29]. For non-randomized studies, we will apply the ROBINS-I, which is designed to assess bias in observational studies [30]. Disagreements will be resolved by discussion; a third reviewer will adjudicate if consensus cannot be reached [18].

### Statistical analysis

#### Effect measures

For dichotomous outcomes, risk ratios (RRs) with 95% confidence intervals (CIs) will be used, with adjusted estimates preferred when available. For continuous outcomes, mean differences (MDs) will be calculated; if measurement scales differ, standardized mean differences (SMDs) will be applied. When only medians with interquartile ranges (IQRs) or ranges are reported, these values will be converted to means and standard deviations (SDs) using validated methods.

#### Eligibility for pooling

Meta-analyses will be conducted when at least three studies report a given outcome. If fewer studies are available or if statistical heterogeneity is excessive, the results will be presented narratively.

#### Meta-analysis model

Meta-analyses will be performed via random–effects models, with restricted maximum likelihood (REML) estimation and Hartung-Knapp-Sidik-Jonkman (HKSJ) adjustment to account for small-sample uncertainty [31].

#### Rare events and zero-event studies

For rare outcomes, generalized linear mixed models (GLMMs) or beta-binomial models will be applied in sensitivity analyses. In zero-event studies, continuity corrections proportional to the treatment arm size or exact methods will be used [32]. Sensitivity analyses will also be performed, excluding such studies.

#### Clustering and repeated cycles

When studies report clustering (e.g., multiple cycles per couple), adjusted estimates will be used preferentially. If unavailable, analyses will be restricted to the first cycle per couple in sensitivity analyses. In multi-arm studies, shared comparator groups will be split, or robust variance estimation (RVE) will be applied to account for statistical dependence [33].

#### Heterogeneity and meta-regression

Between-study heterogeneity will be assessed via τ^2^, *I*^2^ with 95% CIs, and prediction intervals. Heterogeneity will be considered substantial if the *I*^2^ exceeds 75% or if the τ^2^ values are large, as suggested in the established methodological literature [34]. In instances of excessive heterogeneity, quantitative pooling will be deemed inappropriate, and a structured narrative synthesis will be conducted following established best practices [18].

If at least 10 studies are available, meta-regression analyses will be conducted to explore potential sources of heterogeneity. The pre-specified effect modifiers to be investigated include the type of azoospermia (obstructive vs. non-obstructive), site of sperm retrieval (testicular vs. epididymal), use of fresh vs. frozen surgical sperm, embryo stage at transfer, type of embryo transfer (single vs. double), female age, calendar mid-year of study, study design (single-center vs. multicenter), and male factor severity (including endocrine, anatomical, histological, and genetic parameters such as FSH levels, testicular volume, histopathology, and genetic abnormalities such as Klinefelter syndrome or Y-chromosome microdeletions, where reported).

#### Publication bias

Small-study effects will be examined visually via funnel plots and statistically via Egger’s regression test, provided that a minimum of 10 studies is available. Sensitivity analyses will include trim-and-fill methods to evaluate the potential impact of publication bias.

#### Certainty of evidence

The GRADE approach [35] will be used to rate certainty across outcomes (live birth, perinatal, long-term, paternal). The assessment considers five key domains: risk of bias, inconsistency, indirectness, imprecision, and publication bias. Based on these criteria, the certainty of evidence will be categorized as high, moderate, low, or very low. A summary of findings will be prepared for each outcome. In addition, sensitivity analyses restricted to studies with low or moderate risk of bias will be conducted to refine the certainty ratings.

## Discussion

This protocol addresses a critical gap in reproductive medicine. Although ICSI with surgically retrieved sperm has been widely adopted for more than three decades, uncertainty remains regarding its relative efficacy and safety compared with ejaculated sperm, particularly for neonatal outcomes, long-term offspring health, and paternal surgical risks. Previous reviews [15–17] have focused primarily on fertilization and pregnancy, often neglecting live birth and outcomes beyond the perinatal period. Moreover, prior syntheses rarely distinguished OAs from NOAs or considered retrieval sites and preservation methods.

To our knowledge, this will be the first systematic review to comprehensively evaluate reproductive, perinatal, long-term offspring, and paternal outcomes in azoospermic men undergoing ICSI via a prospectively registered, methodologically rigorous approach. By incorporating randomized and non-randomized evidence, applying state-of-the-art risk of bias tools, and grading certainty with GRADE, this review will provide robust conclusions. Subgroup and meta-regression analyses will enhance applicability and help explain heterogeneity.

The implications extend beyond academic interest: the results will directly inform counseling of couples with azoospermia, guide clinical decision-making regarding surgical techniques, and contribute to ART guideline development. By identifying gaps, particularly long-term offspring outcomes and paternal harm, our review will also help prioritize prospective research.

## Supplementary Information

Additional file 1: PRISMA-P and PRISMA 2020 guidelines.

Additional file 2: Example of full search strategy (PubMed).

## Data Availability

Data will be available within the article/supplementary files; full search strategies will be deposited on OSF with DOI‑citable.

## Abbreviations

ART: Assisted reproductive technology
ICSI: Intracytoplasmic sperm injection
OA: Obstructive azoospermia
NOA: Non‑obstructive azoospermia
TESE: Testicular sperm extraction
micro‑TESE: Microdissection TESE
TESA: Testicular sperm aspiration
PESA: Percutaneous epididymal sperm aspiration
MESA: Microsurgical epididymal sperm aspiration
OPU: Oocyte pick‑up
NICU: Neonatal intensive care unit
RCT: Randomized controlled trial
REML: Restricted maximum likelihood
HKSJ: Hartung‑Knapp‑Sidik‑Jonkman
GLMM: Generalized linear mixed model
PRESS: Peer Review of Electronic Search Strategies
CI: Confidence interval
RR: Risk ratio
MD: Mean difference
SMD: Standardized mean difference
IQR: Interquartile range
SD: Standard deviation
RVE: Robust variance estimation
GRADE: Grading of recommendations assessment, development and evaluation
ROBINS-I: Risk of bias in non-randomized studies—of interventions
RoB 2: Revised Cochrane risk-of-bias tool
OSF: Open Science Framework

## References

[1] Esteves SC. Clinical management of infertile men with nonobstructive azoospermia. Asian J Androl. 2015;17(3):459–70. 25677138 10.4103/1008-682X.148719 PMC4430952

[2] Sharma M, Leslie SW. Azoospermia. [Updated 2023 Nov 18]. In: StatPearls [Internet]. Treasure Island (FL): StatPearls Publishing; 2025. PMID: 35201719. Available from: https://www.ncbi.nlm.nih.gov/books/NBK578191/. 35201719

[3] Wosnitzer M, Goldstein M, Hardy MP. Review of azoospermia. Spermatogenesis. 2014;4:e28218. 25105055 10.4161/spmg.28218 PMC4124057

[4] Palermo G, Joris H, Devroey P, Van Steirteghem AC. Pregnancies after intracytoplasmic injection of single spermatozoon into an oocyte. Lancet. 1992;340(8810):17–8. 1351601 10.1016/0140-6736(92)92425-f

[5] Schlegel PN. Testicular sperm extraction: microdissection improves sperm yield with minimal tissue excision. Hum Reprod. 1999;14(1):131–5. 10374109 10.1093/humrep/14.1.131

[6] Lewin J, Lukaszewski T, Sangster P, Williamson E, McEleny K, Al Wattar BH, et al. Reproductive outcomes after surgical sperm retrieval in couples with male factor subfertility: a 10-year retrospective national cohort. Fertil Steril. 2023;119(4):589–95. 36592648 10.1016/j.fertnstert.2022.12.041

[7] Sun X, Yang KL, Zheng QY, Lu QF, Qi ZQ, Liu Y, et al. Effects of different sperm sources on clinical outcomes in intracytoplasmic sperm injection cycles. Andrologia. 2022;54(7):e14438. 35585478 10.1111/and.14438

[8] Du M, Zhang J, Li Z, Liu Y, Wang K, Guan Y. Clinical and neonatal outcomes of children born after ICSI with or without surgically acquired sperm: a retrospective cohort study. Front Endocrinol (Lausanne). 2021;12:788050. 35145477 10.3389/fendo.2021.788050 PMC8823095

[9] Belva F, De Schrijver F, Tournaye H, Liebaers I, Devroey P, Haentjens P, et al. Neonatal outcome of 724 children born after ICSI using non-ejaculated sperm. Hum Reprod. 2011;26(7):1752–8. 21511713 10.1093/humrep/der121

[10] Sadek S, Matitashvili T, Alddin RS, Morshedi B, Ramadan H, Dodani S, et al. IVF outcomes following ICSI cycles using testicular sperm in obstructive (OA) vs. non-obstructive azoospermia (NOA) and the impact of maternal and paternal age: a SART CORS data registry. J Assist Reprod Genet. 2023;40(3):627–37. 36662354 10.1007/s10815-023-02726-x PMC10033785

[11] Sandin S, Nygren KG, Iliadou A, Hultman CM, Reichenberg A. Autism and mental retardation among offspring born after in vitro fertilization. JAMA. 2013;310(1):75–84. 23821091 10.1001/jama.2013.7222

[12] Meijerink AM, Ramos L, Janssen AJ, Maas-van Schaaijk NM, Meissner A, Repping S, et al. Behavioral, cognitive, and motor performance and physical development of five-year-old children who were born after intracytoplasmic sperm injection with the use of testicular sperm. Fertil Steril. 2016;106(7):1673-82.e5. 27793367 10.1016/j.fertnstert.2016.09.011

[13] Norrman E, Petzold M, Bergh C, Wennerholm UB. School performance in children born after ICSI. Hum Reprod. 2020;35(2):340–54. 31957795 10.1093/humrep/dez281 PMC7048711

[14] Yu X, Lu S, Yuan M, Ma G, Li X, Zhang T, et al. Does ICSI outcome in obstructive azoospermia differ according to the origin of retrieved spermatozoa or the cause of epididymal obstruction? A comparative study. Int Urol Nephrol. 2022;54(12):3087–95. 36059025 10.1007/s11255-022-03350-x PMC9606059

[15] Shih KW, Shen PY, Wu CC, Kang YN. Testicular versus percutaneous epididymal sperm aspiration for patients with obstructive azoospermia: a systematic review and meta-analysis. Transl Androl Urol. 2019;8(6):631–40. 32038959 10.21037/tau.2019.11.20 PMC6987592

[16] Catford SR, McLachlan RI, O’Bryan MK, Halliday JL. Long-term follow-up of ICSI-conceived offspring compared with spontaneously conceived offspring: a systematic review of health outcomes beyond the neonatal period. Andrology. 2018;6(5):635–53. 30296010 10.1111/andr.12526

[17] Esteves SC, Agarwal A. Reproductive outcomes, including neonatal data, following sperm injection in men with obstructive and nonobstructive azoospermia: case series and systematic review. Clinics (Sao Paulo). 2013;68 Suppl 1(Suppl 1):141–50. 23503964 10.6061/clinics/2013(Sup01)16 PMC3583175

[18] Higgins JPT, Thomas J, Chandler J, Cumpston M, Li T, Page MJ, et al, editor(s). Cochrane Handbook for Systematic Reviews of Interventions version 6.5 (updated August 2024). Cochrane, 2024. Available from: https://www.cochrane.org/handbook.

[19] Shamseer L, Moher D, Clarke M, Ghersi D, Liberati A, Petticrew M, et al. Preferred reporting items for systematic review and meta-analysis protocols (PRISMA-P) 2015: elaboration and explanation. BMJ. 2015;350:g7647. 25555855 10.1136/bmj.g7647

[20] Page MJ, McKenzie JE, Bossuyt PM, Boutron I, Hoffmann TC, Mulrow CD, et al. The PRISMA 2020 statement: an updated guideline for reporting systematic reviews. BMJ. 2021;372:n71. 33782057 10.1136/bmj.n71 PMC8005924

[21] Zarin DA, Tse T, Williams RJ, Carr S. Trial reporting in ClinicalTrials.gov - the final rule. N Engl J Med. 2016;375(20):1998–2004. 27635471 10.1056/NEJMsr1611785 PMC5225905

[22] Organization WH. International Clinical Trials Registry Platform (ICTRP): mission and vision: World Health Organization; [Available from: https://www.who.int/clinical-trials-registry-platform.

[23] Mahood Q, Van Eerd D, Irvin E. Searching for grey literature for systematic reviews: challenges and benefits. Res Synth Methods. 2014;5(3):221–34. 26052848 10.1002/jrsm.1106

[24] McGowan J, Sampson M, Salzwedel DM, Cogo E, Foerster V, Lefebvre C. PRESS peer review of electronic search strategies: 2015 guideline statement. J Clin Epidemiol. 2016;75:40–6. 27005575 10.1016/j.jclinepi.2016.01.021

[25] Hozo SP, Djulbegovic B, Hozo I. Estimating the mean and variance from the median, range, and the size of a sample. BMC Med Res Methodol. 2005;5:13. 15840177 10.1186/1471-2288-5-13 PMC1097734

[26] Wan X, Wang W, Liu J, Tong T. Estimating the sample mean and standard deviation from the sample size, median, range and/or interquartile range. BMC Med Res Methodol. 2014;14:135. 25524443 10.1186/1471-2288-14-135 PMC4383202

[27] Luo D, Wan X, Liu J, Tong T. Optimally estimating the sample mean from the sample size, median, mid-range, and/or mid-quartile range. Stat Methods Med Res. 2018;27(6):1785–805. 27683581 10.1177/0962280216669183

[28] Shi J, Luo D, Weng H, Zeng XT, Lin L, Chu H, et al. Optimally estimating the sample standard deviation from the five-number summary. Res Synth Methods. 2020;11(5):641–54. 32562361 10.1002/jrsm.1429

[29] Sterne JAC, Savović J, Page MJ, Elbers RG, Blencowe NS, Boutron I, et al. RoB 2: a revised tool for assessing risk of bias in randomised trials. BMJ. 2019;366:l4898. 31462531 10.1136/bmj.l4898

[30] Sterne JA, Hernán MA, Reeves BC, Savović J, Berkman ND, Viswanathan M, et al. ROBINS-I: a tool for assessing risk of bias in non-randomised studies of interventions. BMJ. 2016;355:i4919. 27733354 10.1136/bmj.i4919 PMC5062054

[31] Maruo K, Yamaguchi Y, Noma H, Gosho M. Interpretable inference on the mixed effect model with the Box-Cox transformation. Stat Med. 2017;36(15):2420–34. 28294388 10.1002/sim.7279

[32] Sweeting M, Sutton A, Lambert PC. What to add to nothing? Use and avoidance of continuity corrections in meta-analysis of sparse data. Stat Med. 2004;23(9):1351–75. 15116347 10.1002/sim.1761

[33] Hedges LV, Tipton E, Johnson MC. Robust variance estimation in meta-regression with dependent effect size estimates. Res Synth Methods. 2010;1(1):39–65. 26056092 10.1002/jrsm.5

[34] Higgins JPT, Thompson SG, Deeks JJ, Altman DG. Measuring inconsistency in meta-analyses. BMJ. 2003;327(7414):557–60. 12958120 10.1136/bmj.327.7414.557 PMC192859

[35] Schünemann HJ, Oxman AD, Brozek J, Glasziou P, Jaeschke R, Vist GE, Williams JW Jr, Kunz R, Craig J, Montori VM, Bossuyt P, Guyatt GH; GRADE Working Group. Grading quality of evidence and strength of recommendations for diagnostic tests and strategies. BMJ. 2008;336(7653):1106–10. 10.1136/bmj.39500.677199.AE. Erratum in: BMJ. 2008;336(7654). 10.1136/bmj.a139. Schünemann, A Holger J [corrected to Schünemann, Holger J]. PMID: 18483053; PMCID:PMC2386626. 10.1136/bmj.39500.677199.AE PMC2386626 18483053

