# Supplementary material for: ICSI with surgically retrieved sperm in azoospermia: protocol for a systematic review and meta-analysis of reproductive, perinatal, long-term, and paternal outcomes
Source: Syst Rev. 2025 Dec 10;15:12. doi: 10.1186/s13643-025-03021-9 (PMC12801866; doi:10.1186/s13643-025-03021-9)
Supplement: Supplementary file 2 — Additional file 2: Example of full search strategy (PubMed). [file 13643_2025_3021_MOESM2_ESM.docx]

**Example of full search strategy (PubMed)**

("Azoospermia"[MeSH Terms] OR "Oligospermia"[MeSH Terms] OR ("azoospermi*"[Title/Abstract] OR "NOA"[Title/Abstract] OR "OA"[Title/Abstract] OR "oligosperm*"[Title/Abstract] OR "cryptosperm*"[Title/Abstract] OR "cryptozoosperm*"[Title/Abstract] OR "hypospermatogenes*"[Title/Abstract] OR "low sperm count*"[Title/Abstract] OR "oligoasthenoteratozoosperm*"[Title/Abstract] OR "oligozoosperm*"[Title/Abstract])) AND ("sperm injections, intracytoplasmic"[MeSH Terms] OR ("sperm injection"[Title/Abstract:~3] OR "sperm injections"[Title/Abstract:~3] OR "sperm injected"[Title/Abstract:~3] OR "spermatozoa injection"[Title/Abstract:~3] OR "spermatozoa injections"[Title/Abstract:~3] OR "spermatozoa injected"[Title/Abstract:~3] OR "ICSI"[Title/Abstract] OR "TESE-ICSI"[All Fields] OR "assisted reproductive technolog*"[Title/Abstract] OR "ART"[Title/Abstract] OR "in vitro fertilization"[Title/Abstract] OR "IVF"[Title/Abstract])) AND ("Sperm Retrieval"[MeSH Terms] OR ("MESA"[Title/Abstract] OR "PESA"[Title/Abstract] OR "SRS"[Title/Abstract] OR "SSR"[Title/Abstract] OR "TESE"[Title/Abstract] OR "mTESE"[Title/Abstract] OR "micro TESE"[Title/Abstract] OR "epididymal sperm*"[Title/Abstract] OR "sperm aspiration"[Title/Abstract:~3] OR "sperm extracted"[Title/Abstract:~3] OR "spermatozoa extracted"[Title/Abstract:~3] OR "sperm extraction"[Title/Abstract:~3] OR "spermatozoa extraction"[Title/Abstract:~3] OR "Sperm Retrieval"[Title/Abstract:~3] OR "sperm retrieve"[Title/Abstract:~3] OR "sperm retrieved"[Title/Abstract:~3] OR "spermatozoa retrieval"[Title/Abstract:~3] OR "spermatozoa retrieve"[Title/Abstract:~3] OR "spermatozoa retrieved"[Title/Abstract:~3] OR "surgical sperm"[Title/Abstract:~3] OR "surgically sperm"[Title/Abstract:~3] OR "testicular sperm*"[Title/Abstract]))
